# Supplementary material for: ShapeSpaceExplorer: Analysis of morphological transitions in migrating cells using similarity-based shape space mapping
Source: PLoS Comput Biol. 2026 Jan 5;22(1):e1013864. doi: 10.1371/journal.pcbi.1013864 (PMC12795464; doi:10.1371/journal.pcbi.1013864)
Supplement: S1 Text — (PDF) [file pcbi.1013864.s003.pdf]

ShapeSpaceExplorer: Analysis of morphological transitions in migrating cells using similarity-based shape space mapping

Samuel D.R. Jefferyes, Roswitha Gostner, Laura Cooper, Mohammed M. Abdelsamea, Elly Straube, Nasir Rajpoot, David B.A. Epstein, and Anne Straube

Supplementary Methods

Cell Culture

Human retinal pigment epithelial (RPE1) cells immortalised with hTERT (Clontech) [1] were grown in RPE medium (DM EM/F-12 medium containing 10% FCS, 2.3 g/l sodium bicarbonate, 2mM L-Glutamine, 100 U/ml penicillin and 100 µg/ml streptomycin) at 37°C, 5% CO2 in a humidified incubator. The RPE1 GLA6 cell line was generated by transfecting hT ERT RPE1 cells (Clontech) with mGFP-LifeAct [Riedl et al., 2008] followed by selection with 500 µg/ml Geneticin (Invitrogen). For depletion experiments, small interfering RNA oligonucleotides targeted against Kif1C (5-CCCAUGCCGUCUUUACCAU-[dC]-[dG]-3) or a scrambled control (5-GGACCUGGAGGUCUGCUGU-[dT]-[dT]-3) were transfected using Oligofectamine (Invitrogen) following manufacturer's instructions. Cells were analysed 48 hours after transfection. Depletion efficiency and specificity was validated using immunofluorescence and Western blotting [1].

Live Cell Imaging

A 35mm glass-bottom dishes (Fluorodish) or 2-well chambered coverglass chambers were coated with 10 µg/ml fibronectin (σ). The fibronectin solution was allowed to incubate for 2-12 hours, and was washed twice with ddH2O before equilibrating the chamber with RPE medium. 6000 RPE1 GLA6 cells were seeded into each dish/well and allowed to spread for 4-6 hours. Cell migration experiments were carried out in RPE growth medium in a microscope stage top incubator (Tokai Hit) heated to 37°C and providing 5%CO2. In each experiment, numerous fields of migrating cells were imaged every 5 min for 12 hr using a 20x objective on an Olympus personal Deltavision microscope (Applied Precision, LLC) using a GFP filter set (Chroma) and a Coolsnap HQ camera, controlled by Softworx (Applied Precision, LLC). Frame rate was set at imaging every 5 minutes because this was adequate for tracking purposes, since the cells neither moved nor changed shape suddenly over this time period, and with any faster imaging we would begin to see the cells negatively affected due to photodamage. The resulting images acquired at every time point were 1024x1024 pixels with 322.5 nm/pixel resolution.

Affinity propagation and Seriation

Affinity Propagation [2] is a clustering algorithm that selects a subset of the data to be "exemplars"; all elements are then assigned to exactly one exemplar. Hence, each exemplar forms a cluster with the points that are assigned to it. The algorithm seeks to find the cluster/exemplar configuration that maximises the total sum of exemplar preferences and the similarities between points and their exemplars. This is achieved rapidly by a message passing process that iteratively passes information between nodes

and updates the system. To perform AP clustering on a dataset of size K, the algorithm requires a similarity matrix $\{S_{ij}\}$ and a set of preferences c_k, for all $i, j, k = 1, ..., K$. We set c_k to be constant over k, and equal to the median of $\{S_{ij}\}$. The following messages are computed iteratively:

$$\alpha_{ij} = \begin{cases} c_j + \sum_{k \neq j} \max(0, \rho_{kj}) & \text{if } i = j \\ \min\left[0, c_j + \rho_{jj} + \sum_{k \notin i,j} \max(0, \rho_{kj})\right] & \text{if } i \neq j \end{cases} \quad (1)$$

$$\rho ij = s_{ij} - \max_{k \neq j}(\alpha_{ik} + s_{ik}) \quad (2)$$

where $\alpha_{ij} = 0$ initially. The value of ρ_{ij} can be thought of as a measure of how well suited j is as an exemplar for i, taking into account other potential exemplars for i. The value of α_{ij} can be thought of as a measure of how available j is to serve as the exemplar for i, taking into consideration other points for which j is an exemplar.

On the other hand, Seriation algorithm is an algorithm designed for a package for cluster analysis [3], and it deals with the reordering of branches of a dendrogram in order to optimise the rank order of the corresponding similarity matrix. A dendrogram is a way of illustrating the results of hierarchical clustering [4], but the displayed order of the branches is not considered. In fact there are 2^{n-2} ways of rearranging a dendrogram of n elements. The seriation algorithm chooses an order that optimises the rank order of the similarity matrix, which is a matrix with elements S_{ij} equal to the similarity between elements i and j. We construct a matrix A, corresponding to the rank of each element in a row, *i.e.* in row i let $a_{ij} = 0$ and $a_{ik} = 1$ where $k \neq i$ is the index of the element most similar to element i, and $a_{im} = 2$ where $m \neq i$ is the index of the element next most similar to i and so on. The goal is then to rearrange the rows and columns (symmetrically) to make this rank matrix as close as possible to the perfect rank matrix: Specifically the algorithm tries to minimise the value of

$$\rho = 1 - \frac{\sum_i \sum_j \alpha_{ij} - p_{ij}^2}{n^3 - n} \quad (3)$$

where α_{ij} are the row-wise rank elements as before and p_{ij} are the corresponding perfect rank matrix elements.

Shape space slicing and shape averaging

To visualise how cell morphology changes along the diffusion coordinates, we partitioned shape space into equal slices along each axes separately. For group analysis and shape dynamics, shape space is sliced into grids, slicing both axes simultaneously to result in rectangular shape space slices. The number of slices for X and Y axes is a parameter in the ShapeSpaceExplorer software in both cases. The shapes with diffusion coordinate values within each slice were determined and processed for further analysis. Groups of cell shapes, selected either as an ROI in the interactive user interface or as output of shape space slicing, were visualised as average shape. For each set of curves (scaled to standard path-length and interpolated to uniform parameterisation), we selected one reference curve and then used BAM to align each curve's orientation and cyclic parameterisation with the reference curve. We can then simply find the average shape by computing the mean of all points of each index in all curves.

Region clustering

Self organizing maps (SOM) was used to perform clustering on the projected shape space. The SOM is an unsupervised neural network whose neurons update concurrently

their weights in a self-organizing manner, in such a way that, during the learning process, its neurons evolve adaptively into specific detectors of different input patterns. A basic SOM is composed of an input layer, an output layer, and an intermediate connection layer. The input layer contains a unit for each component of the input vector. The output layer consists of neurons that are typically located either on a 1-D or a 2-D grid, and are fully connected with the units in the input layer. The intermediate connection layer is composed of weights (also called prototypes) connecting the units in the input layer and the neurons in the output layer (in practice, one has one weight vector associated with each output neuron, where the dimension of the weight vector is equal to the dimension of the input).

Shape property measurements

The properties are evaluted using MATLAB *regionprops* and calculated as follows:

- Area: The actual number of pixels in the region.
- Major Axis Length: Scalar specifying the length (in pixels) of the major axis of the ellipse that has the same normalised second central moments as the region.
- Minor Axis Length: Scalar specifying the length (in pixels) of the minor axis of the ellipse that has the same normalised second central moments as the region.
- Eccentricity: Scalar that specifies the eccentricity of the ellipse that has the same second-moments as the region. The eccentricity is the ratio of the distance between the foci of the ellipse and its major axis length. The value is between 0 and 1. (0 and 1 are degenerate cases; an ellipse whose eccentricity is 0 is actually a circle, while an ellipse whose eccentricity is 1 is a line segment.)
- Orientation: The angle (in degrees ranging from -90 to 90 degrees) between the x-axis and the major axis of the ellipse that has the same second-moments as the region.
- Convex Area: Scalar that specifies the number of pixels in the convex hull.
- Solidity: Scalar specifying the proportion of the pixels in the convex hull that are also in the region. Computed as Area/Convex Area.
- Extent: Scalar that specifies the ratio of pixels in the region to pixels in the total bounding box. Computed as the total area divided by the area of the bounding box.
- Perimeter: The distance around the boundary of the region. The perimeter is calculated using the distance between each adjoining pair of pixels around the border of the region.
- Circularity: Measured by computing $P/\left(2\sqrt{\pi A}\right)$ where P is the perimeter and A is the area.
- Symmetry: Scalar specifying the ratio of pixels bounded by both the cell curve and its reflection in its major axis to the number of pixels bounded by the cell curve.
- Max distance from centre: The maximum distance between the centre of mass (of the region bounded by the cell's boundary) and any point on the boundary of the cell.

- Min distance from centre: The minimum distance between the centre of mass (of the region bounded by the cell's boundary) and any point on the boundary of the cell.
- Min/max centre distance ratio: The ratio of the minimum to the maximum distances between the centre of mass and any point on the boundary of the cell.
- Irregularity: Also described as non-circularity, irregularity is computed as $\frac{1+\sqrt{\pi}\max_i \sqrt{(x_i-\overline{x})^2+(y_i-\overline{y})^2}}{\sqrt{Area}} - 1$
- Irregularity 2: This is the irregularity of the negative space of the cell region within the bounding box.

Fourier Descriptors as shape similarity measure

Fourier Descriptors were generated from the cell outlines by performing a Fourier transform, rotation invariance was then gained by taking the absolute value of the Fourier transform (commonly known as the power spectrum). With the curve represented by a discrete sequence $\{x_n\}$, we can make use of the fast Fourier transform

$$X_k = \sum_{n=0}^{N-1} x_n e^{-2\pi ikn/N}, \quad (4)$$

from which we can rapidly compute the power spectrum:

$$P_k = X_k \cdot X_k^*, \quad (5)$$

where X_k^* is the complex conjugate of X_k. These features represent the levels of auto-correlation at different frequencies around the cell's edge. Simply taking the Euclidean distance in this feature space gives us a shape similarity measure.

Retraction and Elongation Speeds

The speeds of the trajectories in shape shape were calculated as the Euclidean distance between consecutive positions of each trajectory. For each step along the trajectory, the angle was also calculated. Steps with angles $0 \pm 45°$ were defined as elongated and steps with angles $180 \pm 45°$ were defined as retracted.

Hidden Markov Model

Eventually, to predict cell behaviour from the constructed cell shape information, we made use of a Hidden Markov Model [5] and considered four hidden states of cell morphology: a depolarised state, a polarised state and the two transition states: depolarising and repolarising.

To implement the Hidden Markov Model, we used the Probabilistic Modelling Toolkit version 3 [6]. This toolbox uses of the Viterbi algorithm [7] to find the most likely states for our data sequences. We trained the model using 19 manually selected image sequences that were deemed typical examples of a turn through depolarisation/repolarisation. The four states were classified manually and the distribution of the shapes in our shape matrix determined.

Angle Checks

We partitioned the track into predicted turn points and the path segments between them. To check a given predicted turn location we examined the subsequence of points in the turn location and the path segments immediately before and after. Let the track subsequence be denoted as $(z_1, z_2, z_3, ..., z_N)$, where each z_i is a planar point (x_i, y_i). Let a and b be the indices within the track subsequence such that for $a \leq i \leq b$, z_i is predicted to be a turn location. Note, in most cases $a = b$, however it is not required. We determined that it was necessary to perform two angle checks for each subsequence, one local check and one distant check, defined as follows. The subsequence passes the distant angle check if the angle between the vectors $(z_a - z_1)$ and $(z_N - z_b)$ is over 25°. The subsequence passes the local angle check if the angle between the vectors $(z_a - \overline{z_a})$ and $(z_b - \overline{z_b})$ is over 40°, where $\overline{z_a}$ is the mean of the set $\{z_i | \max(1, a-10) \leq i \leq \max(1, a-6)\}$ and $\overline{z_b}$ is the mean of the set $\{z_i | \min(N, b+6) \leq i \leq \min(N, b+10)\}$. A turn prediction is marked to be correct if it passes either of these checks. The thresholds were trained using a labelled set of training sequences, each containing one repolarisation-based turn. Track sequences that were identified as curving after or before the turn were excluded from the training of the distant check, track sequences that were identified as delaying after the turn were excluded from the training of the local check.

Straightness Check

We partitioned the track into predicted turn points and the path segments in between them as above. To check for the absence of turns in each path segment we performed a straightness check as follows. We first found the linear fit of the path segment, then computed the perpendicular distance of each point to the fitted line. These perpendicular distances were denoted by $(d_1, d_2, d_3, ..., d_N)$, where N is the number of points in the segment. Then we assessed the straightness by computing

$$D = \frac{1}{L}\sqrt{\sum_{i=1}^{N} d_i^2}$$

where L is the length of perpendicular projection of the whole path segment onto the fitted line. We then say the segment is straight if D is less than 0.175. This threshold was optimised to discriminate a labelled training set of straight and non-straight path segments (all identified as not following a repolarisation mechanism).

Turn Prediction Accuracy

To assess the accuracy of the turn predictions we restricted ourselves to subsequences of cell tracks that contain a turn prediction which passes the angle check surrounded by path segments which pass the straightness check. We defined the true location of the turn to be the point that has the highest perpendicular distance to the straight line that connects the end points of the subsequence. Then we measured the time delay between the predicted turn location and the true turn location. For contrast, we also measured the time delay between the predicted turn location and two other landmarks; the midpoint of the path segment before the true turn location, and the midpoint of the segment after.

References

1. Theisen U, Straube E, Straube A. Directional Persistence of Migrating Cells Requires Kif1C-Mediated Stabilization of Trailing Adhesions. Developmental Cell. 2012;23(6):1153–1166. doi:10.1016/j.devcel.2012.11.005.

2. Frey BJ, Dueck D. Clustering by Passing Messages Between Data Points. Science. 2007;315(5814):972–976. doi:10.1126/science.1136800.

3. Wishart D. Clustan Graphics3 Interactive Graphics for Cluster Analysis. In: Gaul W, Locarek-Junge H, editors. Classification in the Information Age. Springer Berlin Heidelberg; 1999. p. 268–275.

4. Ward JH. Hierarchical Grouping to Optimize an Objective Function. Journal of the American Statistical Association. 1963;58(301):236–244. doi:10.1080/01621459.1963.10500845.

5. Baum LE, Petrie T. Statistical Inference for Probabilistic Functions of Finite State Markov Chains. The Annals of Mathematical Statistics. 1966;37(6):1554–1563.

6. Dunham M, Murphy K. probml/pmtk3; 2023. Available from: `https://github.com/probml/pmtk3`.

7. Viterbi A. Error bounds for convolutional codes and an asymptotically optimum decoding algorithm. IEEE Transactions on Information Theory. 1967;13(2):260–269. doi:10.1109/TIT.1967.1054010.
