# Supplementary material for: ShapeSpaceExplorer: Analysis of morphological transitions in migrating cells using similarity-based shape space mapping
Source: PLoS Comput Biol. 2026 Jan 5;22(1):e1013864. doi: 10.1371/journal.pcbi.1013864 (PMC12795464; doi:10.1371/journal.pcbi.1013864)
Supplement: Attachment — Submitted filename: PLOSCompBiol_Rebuttal_Nov2025_v2.pdf [file pcbi.1013864.s004.pdf]

Response to reviewers' comments:

Reviewer #1: This work involves quantitative representation of shape distributions of migrating mesenchymal cells and analysis of cell morphology during motility. It uses the diffusion maps technique to compare pairs of cell shapes. Using this framework, the authors describe a relationship between cell shape dynamics and cell migration. The need for such a tool to extract shape features of migrating cells is well acknowledged. In that regard, the work presents a software that can be used in experiments to assess cell shape changes in response to perturbations. I think it can also be applied to static cells as well.
Having said that, it is not easy to read - it would help if the methods were described in greater detail. I am not sure if I can clearly see how this work establishes the claimed relationship between cell shape dynamics and migration. The manuscript appears written for readers already well-versed in machine learning terminology, which makes it difficult for those without such a background to follow. It took me quite some time to go through, and I still could not fully understand all aspects. Unfortunately, I do not have access to MATLAB, so I could not see the software in action, which would probably have given me a fairer impression of the work. Some specific comments are below

1. Which types of images does this work on? Does it work for all 2D images, say bright field microscopy, fluorescence microscopy, or also for 3D confocal etc.?

The software can be applied to any 2D outlines of shapes from cells, tissues or inanimate objects – as we mention in the abstract. We include in the software automatic segmentation and tracking for fluorescent image series, but also the option to upload outlines generated either by manually drawing around an object or any other mechanism of segmenting objects. This enables use of the embedding and shape analysis tools even if the inbuilt segmentation algorithm is not suitable. For example, brightfield images of cells don't usually have sufficient contrast for faithful automatic segmentation. 3D images can be used too, but the software would only consider the outline of a 2D projection. There is no requirement to have timelapse images. Shape analysis can be performed on outlines from static images (this is now explicitly mentioned in line 63). We also show the embedding of a standard shape set that did not have temporal information as Supplementary Figure S1.

2. Even though the definition of the shape similarity using BAM is quite intuitive, it will be good to show how good the BAM metric is by showing it on some standardized contours. This will also help justify the use of the optimal number of nodes (512).

We have included a figure where we embedded standardised shapes from a public dataset (https://github.com/2dshapesstructure). This shows that BAM can separate the different shape classes successfully and order them into a map. We show these data in new supplementary Figure S1.

Our cell shapes were segmented from microscopy images at pixel resolution. Depending on the cell area and number of protrusions, the number of original nodes varied. In our untreated dataset of 37818 cell outlines, the number of nodes was 364 ± 118 (mean ± SD) with the maximum at 1139. We say that 512 nodes were sufficient as we could find virtually no deviation in BAM distances if calculating these for data extrapolated to 2046 nodes compared to 512 nodes. The optimal number of nodes for this dataset might be lower if optimising for speed as there was also minimal information loss if using 256 nodes. The standard set we analysed as an example above are described with 102 to 188 nodes per shape, suggesting that we are considering sufficient number of points to describe even highly complex shapes without risking a loss of information.

3. The context of the use of the Diffusion Map embedding is not clear to me. Is it to represent all the cell shape data (from a sequence of cell images) in a low dimensional form? For example, it will help if it is explicitly states what does x and y represent in equation (3) in this context.

Yes, exactly, the Diffusion Map embedding is a low dimensional representation of the distances between all shapes with the axes optimised to capture the largest variations in shape for any given dataset. It represents all shapes in the dataset, including, if provided as input, sequences of cell shapes. We are now including an author summary and more accessible overview of the approach in lines 35 onwards.

We changed equation 3 and now use the description for the BAM distance between two curves υ and ν consistent with equations 1 and 2. We also extended the text to clarify all variables used. See lines 108-110.

4. Given the closed loop nature of the cell contours, why can't Fourier series representation be used as a low-dimensional representation?

Fourier Descriptors can be used and others have implemented tools using these as mentioned in the introduction (lines 17-20). However, we believe that BAM is superior as shape descriptor for 2D shapes.

To compared the performance, we calculated the Fourier Descriptors and then took the Euclidean Distance in this feature space as alternative shape similarity measure to BAM. Calculating the Fourier Descriptor distance was about 4.5 time faster than BAM. However, comparing the performance qualitatively by using both approaches to identify the 5 closest cell shapes in the dataset to one randomly selected example, shows that in several cases BAM seems to perform better and consistently picks out shapes with characteristic features. For example, in line 5, all BAM-selected shapes have a thin tail on a D-shaped cell, and in line 8, all BAM-selected shapes have a wider front and the cell bends to the left. We believe that this is because some aspects of shape information is lost when the phase information is removed during calculating the Fourier transform power spectrum. Furthermore, the shape space embedding using BAM is reversable, allowing the generating of synthetic contours from arbitrary points in our low-dimensional representation. Because Fourier Descriptors map into the space of power spectra, and the power spectra are missing the phase information, an attempt on reversal will not lead to a unique solution.

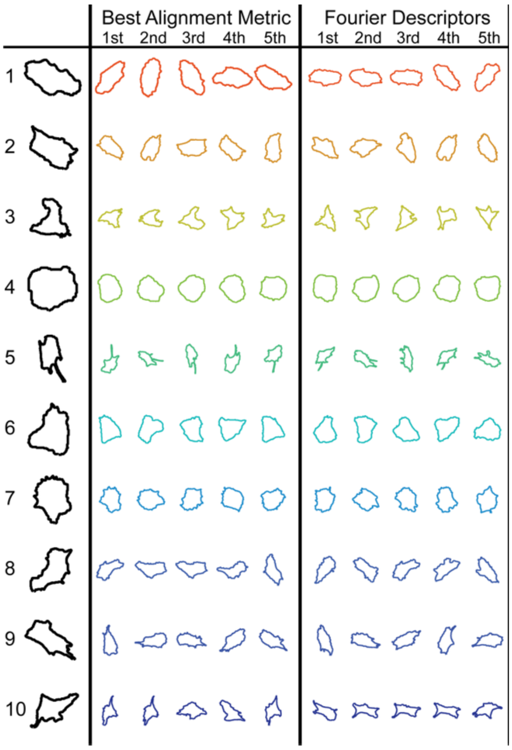

We now describe this in the text (lines 179-187) and the data in new Figure 2, which also contains exemplars from hierarchical clustering plotted directly at their position in shape space to aid visualisation of where to find cells with different morphology.

5. I also could not understand the motivation behind the calculation of pairwise alignment for all shapes (lines 115-116).

The pairwise alignment is the core of BAM. It allows us to determine the magnitude of shape differences between pairs of shapes and therefore the distance in shape space. If we were to calculate the difference in shape prior to alignment, two identical non-circular shapes would give very different distances depending on their relative orientation. Therefore, we minimise overlap between two shapes before we calculate the sum of their differences as a measure of how distant these two shapes are. We are now explaining the overall strategy in the author summary and the start of the implementation section (starting in line 36, which hopefully clarifies the need for pairwise alignment and distance measurement).

6. Shouldn't similarity be calculated between consecutive shapes?

We calculate similarity (or differences) between all shapes in the dataset. That includes the similarity between consecutive shapes. Without including all other shapes, it is not possible to plot all shapes and their trajectories together in a single shape map. As above, we hope that the principle of the approach is now more clear.

7. It is not clear to me what exactly is meant by the "shape space splicing" (line 129).

It is shape space slicing – we cut the space into strips and rectangles defined by user input of how many slices they would like in X and Y (i.e. diffusion coordinate 1 and diffusion coordinate 2). We then determine which shapes fall into which slice and perform downstream analysis with all members of a slice. We use this is in Figures 3a, 4a-b and 5b. In 3A, slicing along the diffusion coordinates is used to visualise the main features changing along each axis by calculating the average shape in each slice. In 4a-b, it is used for counting the number of shapes in each section of a 4-by-2 grid for group analysis. In 5b, we use it to show the distribution of vectors pointing from shapes in each area of an 8-by-4 grid to their consecutive shape in the time series. In all cases, the slices separate the shape space into regions. All shapes in this region are aligned to each other to calculate the average shape, are counted to report relative abundance or dynamic features are extracted such as the direction or speed (in shape space) to the next consecutive shape. We added a section in the supplementary material to explain how we do shape space slicing.

8. Table 1 describes the physical characteristics of the cell along with diffusion map coordinates. It will help the reader if this is discussed in little more detail. How do we relate the DC_i's with physical characteristics?

Figure 3a shows the average shapes along the first two DC axes using the slicing tool (see point 7). This allows a visual understanding of the physical characteristics along the axes. We describe this in lines 200-205 now more extensively.

Furthermore, table 1 shows correlation with commonly used shape features. It is clear that there is no single classical shape measure that captures full range of physical characteristics along the DC axes, but several of the shape features change gradually diagonally through shape space as can be seen in Figure 3b.

We have expanded the text to explain what the diffusion coordinates mean in more detail again when we compare them to the different shape features (lines 216 to 221).

9. Equation (4) calculates Pearson correlation. Are all the curves independent? Will the independence or dependence affect the correlation analysis?

The cell outlines in the dataset are not completely independent of each other as we analyse time series. However, this does not affect the correlation analysis here as where we compare the value for a shape feature with the corresponding value for each diffusion coordinate for any given curve, rather than the correlation between different curves. We changed the wording in lines 211-213 slightly to explain the variables more clearly.

10. I am afraid I do not find it apparent from the figure "and significantly fewer highly elongated cells with long tails were present in the siKif1C dataset compared to control cells". (line 219)

The elongated cells with long tails are in region 4 both in new Figure 4a-b and 4c-e. We have now added the average shape per region in Fig. 4b to not require the reader to check back in Figures 2 and 3 where each shape can be found and also refer to regions (1 for round and slightly elongated cells and 4 for highly elongated cells with tail) in the text in lines 247-250. We hope that makes it more obvious where elongated cells with tails are found in the dataset.

11. In Fig. 3, are untreated cells same as control?

We show three experimental conditions in previous Figure 3, now Figure 4. The untreated dataset that has been introduced in Figures 1-2 and used to build the diffusion map, is shown as grey dots in the background. In Figure 3, we include as out-of-sample-extension a dataset of siRNA-treated cells. The dataset uses cells treated with a non-targeting control siRNA in addition to cells treated with the siRNA targeting KIF1C. The latter two have been imaged side-by-side in the same overnight experiment. It is expected that siControl has very little effect on cell shape compared to untreated cells, but variations in experimental condition such as glass coating, passage number etc. could result in morphological changes. Therefore, it makes sense to compare internal controls when making quantitative measurements as we present in Figures 4 and 5c. The treatments are mentioned in the main text in lines 237-240 and described in the cell culture section in the supplementary material.

12a. Again, I do not follow the detection of "cell turning". Can't that be seen directly from the trajectory itself?

Yes, cell turning can be seen from the trajectory in real space and we use this here as the ground truth to compare to our prediction from shape information alone. As we show that it is possible to predict cell turning from shape dynamics, it implies that other cell behaviour can be analysed too. One can imagine expanding the tool to analyse both the real space trajectory and cell shape to allow the unsupervised analysis of how cells turn, how shape relates to speed and directionality of cell migration in large datasets with different treatments. We have now discussed some of this in the Future Directions section starting from line 346.

12b. In Fig. 4 (a), it will help if the direction of the flow can be highlighted.

We have now added arrows at the end of some of the track segments to indicate directions, new Figure 5a. However, the best approach to look at flows is to either inspect individual trajectories within the interactive software (as in Fig. 1g, also see software documentation for an example here: https://cmcb-warwick.github.io/ShapeSpaceExplorer/display_shape_features/) or to look at the general direction of flow in each section of shape space using Rose diagrams as shown in Fig. 5b for an 8-by-4 grid and described in the documentation here: https://cmcb-warwick.github.io/ShapeSpaceExplorer/shape_space_dynamic/#sliced-shape-space-average-dynamics .

Reviewer #2: In this manuscript, the authors present a software (MATLAB-based Graphical User Interface) for the analysis of cell shapes and migration types. The method of analysis is based on a similarity matrix that quantifies the overall proximity of uniformly distributed points representing individual cell outlines. The software utilizes an effective optimization approach to calculating the similarity matrix, diffusion map embedding, unsupervised hierarchical clustering, and even a Hidden Markov Model approach for characterization of state transitions during cell migration. The manuscript is well-structured, written with care, and substantiated with comparative analyses and applications to experimental data. This software will be of interest to both experimental and computational cell biologists. I do not have any major concerns, but I do have some suggestions to consider during the revision stage.

Minor concerns/suggestions:

1. Throughout the paper, the authors provide their choices of some parameters (usually 4 or 5) without much justification. For example, "distinct cell IDs for each continuous track segment of at least 5 frames length", "5 eigenvectors were found to be sufficient", "we have selected K = 5 for accurate and fast assignment". I don't have anything against number 5, and I understand that some choices need to be made, but at least in some cases, the authors could run analyses with 4, 5, and 6 to show that the results don't change or that the accuracy plateaus. This could go to Supplemental Materials. Alternatively, if the software provides an easy way to adjust these parameters and interactively see results, the authors should clearly state that in the text where they make such choices.

While we used 5 frames as cutoff for our dataset, minimal track length is a filter parameter in the segmentation/tracking algorithm as described in the documentation (https://cmcb-warwick.github.io/ShapeSpaceExplorer/cell_segmentation/#filter-out-shapes-with-small-area-size). The minimal length should not affect the results described in the paper. Obviously, the analysis of cell turns requires much longer cell tracks, but morphology could be analysed from still images also. Therefore, users can decide whether they want to filter their data for a minimal track length of their choice or for example analyse individual shapes only. This is mentioned in the text in lines 61-63.

We calculate 5 eigenvectors but only use the first 2 eigenvectors (or diffusion coordinates) for visualisation because they capture the vast majority (86%) of variation in our RPE cell dataset as shown in Figure 1d. Also, Table 1 shows that diffusion coordinates 3, 4 and 5 show no higher correlation with any shape feature than diffusion coordinates 1 or 2 and therefore are unlikely containing a critical aspect not yet covered in the 2D shape space. Therefore, we provided already 3 spares as default within the software if people wish to analyse their data using 3, 4 or 5 coordinates. However, we have now modified the software to include the number of determined diffusion coordinates as a variable, with a minimum of 2.

We have chosen 5 as the number of nearest neighbours for positioning new shapes into an existing shape space during out-of-sample-extension. We have implemented two methods of out-of-sample extension (K-Nearest-Neighbours described and used for the data in the paper; and Laplacian Pyramids) and the user can choose which to use. We have tested 3 and 5 nearest neighbours and 5 got a more comparable result to the Laplacian Pyramid embedding. K is now also a user-defined variable in the software as shown in the software documentation here: https://cmcb-warwick.github.io/ShapeSpaceExplorer/out_of_sample_extension/. We now mention both methods and the user-defined variable in the text in lines 132-135.

2. In Figure 4a, the authors illustrated cell tracks in shape space coordinates. I wonder if it is possible to classify these tracks (based on the characteristics of the tracks in the real and/or shape space). The authors opted to consider in detail only cell turning, which is interesting and valuable, but some unsupervised clustering or cell trajectories could also be important for broader applications, at least as the first step.

Upon visual inspection, we have not observed high density paths taken by cells in shape space as we had initially hoped. However, we show the general flow in 5b that indicates primarily parallel trajectories in shape space.

It is possible to deeper analyse and classify trajectories in shape space and we implemented some useful tools already that are not described in the paper. However, our detailed documentation on github includes all features. The shape dynamics section (https://cmcb-warwick.github.io/ShapeSpaceExplorer/shape_space_dynamic/) outlines currently implemented features. This includes the separation of shape space into regions and selecting all trajectories that go from one region to another. This can be used to measure shape change speed between any two selected subregions of shape space (we briefly mention this now in line 51-52). Also, dynamics data for all shapes in a user-defined slice of shape space are output into a data structure and saved as a DynamicData.mat file for further user-defined analysis. Of these, the direction towards the consecutive shape is plotted as Rose diagram as shown in Fig. 5b.

We added some ideas on track classification or segmentation into the futures direction section as we felt it would be out of scope for the current manuscript to add this. This can be found starting in line 346.

3. On Page 11, near the end of the first paragraph, there seems to be an incomplete sentence: “We find that more than 61% of predicted turn points were within the 5 closest points (±10 min) to the true turn, while only 14% of the control points were (Fig. 6g).”

We now added “... close to a turn” to make the sentence clearer. This can now be found in line 309.

4. The last section is titled “Availability and Future Directions”, but I can’t find anything about “Future Directions”. Either the title or the text needs to be corrected.

This is a fair point. The heading is part of the template, so inspired by some of the suggestions from both reviewers we now propose future developments with regards to additional flow analysis and combined analysis of shape dynamics and real space trajectories. This is in lines 346-371.
